# Glia-Neuron Interactions in the Retina Can Be Studied in Cocultures of Müller Cells and Retinal Ganglion Cells

**DOI:** 10.1155/2016/1087647

**Published:** 2016-06-26

**Authors:** D. M. Skytt, A. K. Toft-Kehler, C. T. Brændstrup, S. Cejvanovic, I. S. Gurubaran, L. H. Bergersen, M. Kolko

**Affiliations:** ^1^Department of Neuroscience and Pharmacology, Faculty of Health and Medical Sciences, University of Copenhagen, Blegdamsvej 3b, 2200 Copenhagen, Denmark; ^2^Brain and Muscle Energy Group, Faculty of Dentistry, Institute of Oral Biology, University of Oslo, P.O. Box 1152, Blindern, 0316 Oslo, Norway; ^3^Center of Healthy Aging, Department of Cellular and Molecular Medicine, University of Copenhagen, 2200 Copenhagen, Denmark; ^4^Zealand University Hospital, Department of Ophthalmology, Vestermarksvej 23, 4000 Roskilde, Denmark

## Abstract

Glia-neuron partnership is important for inner retinal homeostasis and any disturbances may result in retinal ganglion cell (RGC) death. Müller cells support RGCs with essential functions such as removing excess glutamate and providing energy sources. The aim was to explore the impact of Müller cells on RGC survival. To investigate the Müller cell/RGC interactions we developed a coculture model, in which primary Müller cells were grown in inserts on top of pure primary RGC cultures. The impact of starvation and mitochondrial inhibition on the Müller cell ability to protect RGCs was studied. Moreover, the ability of Müller cells to remove glutamate from the extracellular space was investigated. RGC survival was evaluated by cell viability assays and glutamate uptake was assessed by kinetic uptake assays. We demonstrated a significantly increased RGC survival in presence of untreated and prestarved Müller cells. Additionally, prestarved Müller cells significantly increased RGC survival after mitochondrial inhibition. Finally, we revealed a significantly increased ability to take up glutamate in starved Müller cells. Overall, our study confirms essential roles of Müller cells in RGC survival. We suggest that targeting Müller cell function could have potential for future treatment strategies to prevent blinding neurodegenerative retinal diseases.

## 1. Introduction

Interactions between the most inner retinal neurons, the retinal ganglion cells (RGCs), and the most abundant retinal glial cells, the Müller cells, are essential to a functional retinal homeostasis. Müller cells span the entire thickness of the retina from the inner nerve fiber layer near the vitreous to the outer segment near the retinal pigment epithelium. The Müller cells are specialized radial glial cells and constitute an anatomical and functional link between neurons and the cellular environment such as blood vessels, the vitreous chamber, and subretinal space. They play a pivotal role in maintaining the structural integrity of the retina as well as sustaining the retinal homeostasis by participating in essential processes such as glucose metabolism, substrate exchange, and vascular regulation [1, 2].

Virtually every aspect of inner retinal homeostasis and function involves a glia-neuron partnership. Growing evidence supports this particular interaction as being fundamental for different aspects of neurodegenerative retinal diseases [2–4]. However, the present knowledge about the partnership between RGCs and Müller cells is limited. The pathological mechanisms of neurodegenerative diseases in the retina are still being debated and there are various hypotheses concerning the cause of the RGC death. Particularly glutamate excitotoxicity [5–8], mitochondrial dysfunction [9–12], oxidative stress [9, 13, 14], disturbed energy metabolism [15–18], altered autoregulation [19, 20], and finally sparse studies on disturbed Müller cell function [3, 5, 15] are among the discussed precursors of RGC death.

The most abundant excitatory neurotransmitter in the central nervous system, including the retina, is the amino acid glutamate [21]. Glutamate is taken up by glutamate transporters into the Müller cells and hence the glutamate transporters are ultimately responsible for balancing the extracellular glutamate level between physiological signalling and pathological overactivation. In Müller cells the predominant glutamate transporter is the excitatory amino acid transporter 1 (EAAT1, also known as GLAST) [22, 23]. We have previously reported that cell cultures of the human Müller glia cell line, MIO-M1 [24], are capable of increasing their glutamate uptake and their expression of EAAT1 during starvation [15], thus indicating a regulatory mechanism to prevent excitotoxicity of the RGCs.

Previous studies have reported improved survival of RGCs cultured with retinal glia cells [5, 25–28]. To the best of our knowledge there have been no studies examining the consequences of energy starvation on the Müller cell ability to promote RGC survival. Here, we describe a coculture model to study the glia-neuron interaction. We explore the effects of prestarvation and starvation on survival of primary Müller cells and primary RGCs. Furthermore, we examine the effect of starvation and mitochondrial inhibition on primary Müller cell viability and primary RGC viability. Finally, we investigate the capacity of glutamate uptake in Müller cells during starvation.

Our study provides knowledge of interactions between primary RGCs and primary Müller cells in a coculture system. We show a significant increase in RGC survival in presence of Müller cells. A significant Müller cell protection is found in both untreated cocultures as well as in prestarved cocultures and in prestarved cocultures with inhibited mitochondrial function. Finally, we demonstrate an increased capacity of Müller cells to transport glutamate during starvation. Overall, our study suggests a vital role of Müller cells in the RGC survival.

## 2. Materials and Methods

### 2.1. Primary Cell Cultures

Primary Müller cells and primary retinal ganglion cells were cultured from dissected retinas of neonatal mice (C57Bl/6J, Charles River, Germany) at postnatal day 6–8 or 5, respectively. The mice were sacrificed by cervical dislocation and the eyes were enucleated into D-PBS. Retinas were carefully dissected under a microscope (Leica S4E).

### 2.2. RGC Purification

Cultures of primary RGCs were purified by sequential immunopanning as described by the group of Professor Barres [29, 30]. Briefly, dissected retinas were digested with papain at 37°C for 45 minutes, which was terminated by rinsing the cells in buffers containing increasing concentrations of ovomucoid (20–40 mg/mL). Following a gentle trituration, the retinal cells were resuspended in panning buffer containing insulin (5 *μ*g/mL), filtered through a 20 *μ*m nylon mesh, and then incubated with rabbit anti-macrophage antibody (AIA51240, Accurate Chemical, USA). Retinal cells were incubated sequentially on three immunopanning dishes: the first two coated with anti-rabbit secondary antibodies (goat anti-rabbit IgG, 611-1102, Rockland, USA) and the third with a RGC specific Thy-1 antibody (LS-C14009, LifeSpan, USA). The Thy-1 plate was rinsed multiple times with EBSS. RGCs were released from the final panning dish with trypsin (Sigma-Aldrich, USA) and the trypsinization was terminated using Neurobasal medium (12349-015, Gibco by Life Technologies, USA) containing 33% fetal bovine serum (Biological Industries, Israel). RGCs were plated at high density (150.000 cells/cm^2^) on poly-D-lysine (10 *μ*L/mL, Sigma-Aldrich) and laminin (10 *μ*g/mL, Sigma-Aldrich) coated cell culture plates (TPP, Switzerland). The RGCs were kept in culture in a 37°C humidified atmosphere with 10% CO_2_ for 6 days in ND-growth medium with change of a third of the medium volume on the third day in culture. The ND-growth culture medium is formulated from Neurobasal medium mixed in equal amounts with DMEM high-glucose medium (31966, Gibco) with addition of penicillin (1000 units/mL), streptomycin (1000 *μ*g/mL), insulin (0.5 *μ*g/mL), sodium pyruvate (1.5 mM), 3,3′,5-Triiodo-L-thyronine (40 ng/mL), L-glutamine (3.7 mM), B27 supplement (1x, Gibco), and trace elements B (1x, Cellgro, USA). The medium was supplemented with a Sato stock consisting of transferrin (100 *μ*g/mL), BSA (100 *μ*g/mL), progesterone (0.63 ng/mL), putrescine (16 *μ*g/mL), and sodium selenite (40 ng/*μ*L). Finally, we added NAC (5 *μ*g/mL), biotin (10 ng/mL), BDNF (50 ng/mL, 248-BD-025, R&D Systems, UK), CNTF (10 ng/mL, 257-NT-010, R&D Systems), FGFb (10 ng/mL, PMG0034, Gibco), and forskolin (4,2 *μ*g/mL) to the medium.

### 2.3. Müller Cell Purification

The Müller cells were cultured essentially as described by Hicks and Courtois [31]. Briefly, the dissected retinas were incubated for 30 minutes in DMEM (21885, Gibco) supplemented with collagenase (65 U/mL) and trypsin (0.25%). Afterwards, the cells were rinsed twice in DMEM containing 10% fetal bovine serum (Gibco) to terminate the digestion. The digested retinas were mechanically dissociated by gentle trituration through a 14 G needle producing a suspension of tissue microaggregates and single cells. Cells were seeded in tissue culture plates or flasks (TPP) or on 0.4 *μ*m membrane inserts (Falcon, USA) at a density of 0.6 retina/cm^2^. Cells were maintained at 37°C in a humidified atmosphere with 5% CO_2_ cultured in DMEM containing glucose (1.0 g/L), pyruvate (1 mM), L-alanyl-glutamine (3.97 mM), supplemented with penicillin (90 Units/mL) and streptomycin (90 *μ*g/mL), and 10% fetal bovine serum (Gibco). The cells were kept in culture for 10–14 days with culture medium changed twice a week. Aggregates and debris were removed by forcible resuspension of medium onto the cell monolayer. Cell cultures of approximately 80–90% confluency were used for experiments.

### 2.4. Immunocytochemistry

Immunocytochemistry was performed on cultures of primary Müller cells cultured for 10 days on poly-D-lysine coated cover glass slides and primary retinal ganglion cells cultured on poly-D-lysine/laminin coated cover glass slides for 6 days. The cells were normal conditioned before the fixation. Briefly, the cells were fixed with 4% paraformaldehyde for 20 minutes at room temperature and the membranes were permeated using 0.02 mol/L glycine and 0.1% Triton-X-100 in PBS for 30 minutes at room temperature. Unspecific binding sites were blocked using 3% freshly diluted BSA (A8806, Sigma-Aldrich) for 1 hour at 4°C. Cell cultures were immunostained using a primary antibody against either glutamine synthetase (GS, G2781, Sigma-Aldrich, 1 : 200), excitatory amino acid transporter 1 (EAAT1, AB416, Abcam, UK, 1 : 200), microtubule associated protein 2 (MAP2, M4403, Sigma-Aldrich, 1 : 500), or growth associated protein-43 (GAP-43, AB5220, Millipore, USA, 1 : 500) combined with Alexa Fluor 488 (A11034, Invitrogen, USA) or Texas Red (T862, Invitrogen) as secondary antibody. On some slides nuclei were counterstained with 0.3 *μ*mol/L DAPI (D3571, Invitrogen) for 3 minutes. Negative controls were treated without primary antibody. The slides were mounted with fluorescent mounting medium (Dako, Denmark) and sealed with nail polish. For RGCs, images were obtained using an iMIC confocal microscope (Till Photonics, FEI, Germany) equipped with appropriate filter settings for detecting DAPI, CY2/Alexa488, and Texas Red/Alexa561/594. The iMIC was equipped with the following objectives: ×20, numerical aperture (NA) = 0.75. The images of the cell culture were stitched together using the LA Stitch plug-in in Fiji software (version 1.47q, NIH, USA) to create an image of the entire culture. For Müller cells, fluorescence microscopy was performed using LEICA DMIL LED equipped with filter cubes (DAPI, GFP ET, and TX2 ET) and Leica Application Suite V4. Pictures were captured using Leica DFC420 C Digital Microscope Camera and analysed using Photoshop CS6.

### 2.5. Experimental Incubations

Our study included different types of media for different types of experiments.* Müller Cells*. A DMEM medium (A14430, Gibco) with a formulation of amino acids and typical extracellular concentrations of inorganic salts was used for incubations of monocultures of Müller cells. In the control incubations the DMEM was added 6 mM glucose, 1 mM pyruvate, and 2.5 mM glutamine. Prestarvation or starvation of Müller cells was performed by incubation for 24 hours in the DMEM by omitting glucose, pyruvate, and glutamine. All incubations of monocultures with Müller cells were performed at 37°C and 5% CO_2_.* RGCs*. For monocultures of RGCs and cocultures of RGCs and Müller cells, the control medium consisted of the ND-growth medium detailed in the methods section above for RGC culturing (containing 25 mM glucose, 1.5 mM pyruvate, and 3.7 mM glutamine). For the starvation experiments the Neurobasal (05-0128DJ, Gibco) and DMEM (A14430, Gibco) components of the ND-growth medium were replaced with glucose-, pyruvate-, and glutamine-free alternatives with otherwise identical compositions. All incubations involving RGCs were at 37°C and 10% CO_2_. To block mitochondrial activity 10 *μ*M antimycin A was added to the incubation medium.

### 2.6. LDH Toxicity Assays

Lactate dehydrogenase (LDH) cell cytotoxicity kit (Takara Bio Inc., Japan) was used to quantitatively assess cell survival. The medium was changed and the cells were starved (in absence of glucose, glutamine, and pyruvate), treated with 10 *μ*M antimycin A, or left untreated (control) for 24 hours. Plates were centrifuged at 500 RPM for 10 minutes, and the supernatants were used for LDH determination as a measure of cell viability. The LDH yield participates in a coupled reaction that converts yellow tetrazolium salts into a red formazan product, which was measured as a 490 nm absorbance reading. Each LDH analysis was performed in triplicate and corrected on single sample/well basis and correlated to total cell death by Triton-X in each sample/well.

### 2.7. MTT Viability Assay

As a second viability assay, Müller cell survival was determined by the colorimetric method, MTT (3-(4,5-dimethyl-2-thiazolyl)-2,5-diphenyl-2H-tetrazolium bromide), measuring the ability of viable cells to reduce MTT to a purple formazan salt, which can be measured at absorbance 560 nm. The Müller cell cultures were incubated in either starvation DMEM or starvation DMEM treated with 1 mM glutamate or left untreated (control DMEM) for 24 hours. Subsequently, the cells were incubated with 12 mM MTT solution for one hour at 37°C and the cells were solubilized in 10% SDS and 0.01 M HCl. Eighteen hours later, the absorbance at 560 nm was measured. The background readings (blank wells with medium, MTT solution, and solubilization buffer) were subtracted from the absorbance readings of the treated wells to obtain an adjusted absorbance reading representing cell viability. Finally, the readings were divided by the adjusted absorbance readings of untreated (control) cells to obtain a percentage of cell survival.

### 2.8. Glutamate Uptake Assay

The kinetic characterization of glutamate transport into the Müller cells was performed as described in our previous study [15]. Primary Müller cells were cultured in 24-well plates and cells were treated with the relevant conditions for 24 hours. The cells were preincubated for 3 minutes at 37°C in HBSS (HEPES buffered saline solution; 142 mM NaCl, 5 mM KCl, 1 mM CaCl_2_, 1 mM MgCl_2_, 1 mM Na_2_HPO_4_, and 10 mM HEPES) containing different concentration of L-glutamate (range 1–500 *μ*M). Subsequently the Müller cells incubated for additionally 3 minutes in HBSS including trace amounts (1 : 200) of L-[3,4-3H]-glutamate (4 *μ*Ci/mL, Perkin-Elmer, Waltham, MA). The cellular content of radioactivity was determined in cells extracted in KOH and analysed by liquid scintillation counting in a Beckman LS6500 Liquid Scintillation Counter (Beckman Coulter, USA). Uptake rates were corrected for protein concentrations, which were measured in the cell extracts using BCA protein assay kit (Sigma-Aldrich). Unspecific uptake was determined by incubation at 0°C. Uptake kinetics was assumed to follow the normal Michaelis-Menten kinetics. The maximal uptake rate, *V*_max_, was determined using a nonlinear regression in GraphPad Prism 6 (*y* = *V*_max_ · *x*/(*K*_*m*_ + *x*)).

### 2.9. Cocultures of RGCs and Müller Cells

To investigate the glia-neuron interactions, cocultures of RGCs and Müller cells were established. Primary Müller cells were precultured in Falcon 0.4 *μ*m membrane inserts (Corning, USA) for 9 days in the DMEM used for normal cell culturing as described above. Likewise RGCs were cultured in coated 24-well plates for 5 days as detailed previously. When assembling the coculture, a complete medium change was performed for both RGCs and Müller cells with ND-growth medium. Subsequently, the inserts with Müller cells were placed into the wells containing RGCs, and the cocultures were maintained in a 37°C humidified atmosphere with 10% CO_2_ for 24 hours to examine cell viability following the conditions detailed above. To measure cell viability in cocultures media were collected for LDH analysis.

### 2.10. Statistical Analysis

All statistical analyses were performed in GraphPad Prism 6.0 software (GraphPad Software, USA) and *P* values less than 0.05 were considered significant. The sample size of each experiment (*n*) has been determined from either a triplicate or more. All data are expressed as means ± SEM and differences between conditions were analysed using unpaired two-tailed *t* tests when comparing two sets of data. If more groups of data were analysed, one-way analysis of variance (ANOVA) followed by Tukey's multiple comparison test was performed.

## 3. Results

### 3.1. Characterization of Primary Cell Cultures

**Figure 1:**
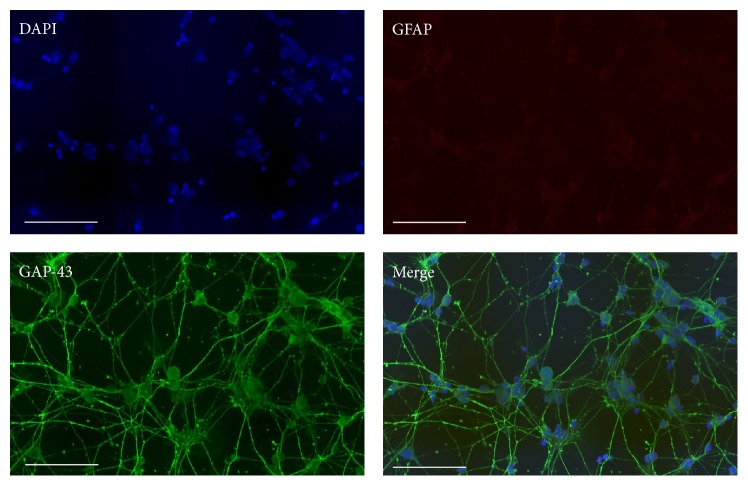
Immunocytochemical staining of primary mouse RGCs. The purity of the primary RGCs was verified by positive immunostaining with GAP-43 (green) surrounding nuclei stained with DAPI (blue). The RGCs lacked GFAP (red) staining confirming the absence of activated macroglial cells in the culture. Scale bars represent 100 *μ*M.

**Figure 2:**
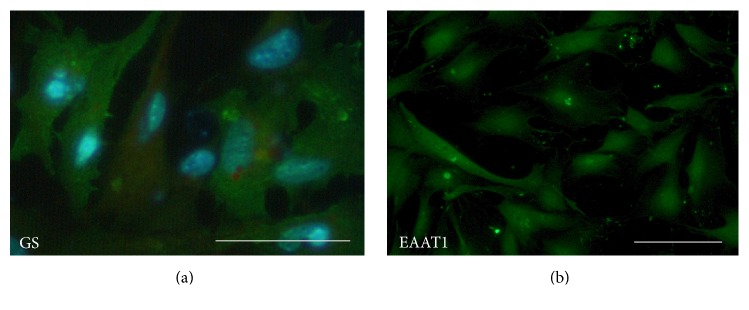
Immunocytochemical staining of primary mouse Müller cells. Primary Müller cells positively expressed the Müller cell specific marker, glutamine synthetase (GS) (GS green and DAPI stained nuclei blue, (a)). In addition, Müller cells expressed the glial cell specific glutamate transporter EAAT1 (EAAT1 green, (b)). All scale bars represent 100 *μ*M.

To positively characterize the primary cell cultures as Müller cells and RGCs we performed immunocytochemical staining. During isolation of RGCs by the immunopanning technique we found some variability of the RGC homogeneity and to evaluate the purity of the isolation we performed immunocytochemical staining of RGCs. The staining confirmed the neuronal origin of the RGCs by demonstrating expression of GAP-43 (green, Figure 1) surrounding all nuclei stained with DAPI (blue, Figure 1). In contrast, the lack of GFAP staining in the cultures confirmed the absence of reactive macroglia. During some RGC preparations, we observed a degree of contamination by other cell types in the RGC cultures, probably from insufficient rinsing of the Thy-1 immunoplates. In such case, the prepared cells were discharged. The immunofluorescence labelling confirmed the expression of glutamine synthetase (GS) in the cytosol of the Müller cells (Figure 2(a)), which in the retina is selectively expressed in Müller cells [32]. The primary Müller cells also stained positive for the major glutamate transporter EAAT1 (Figure 2(b)).

### 3.2. Starvation and Simultaneous Mitochondrial Inhibition Decrease RGC and Müller Cell Survival

**Figure 3:**
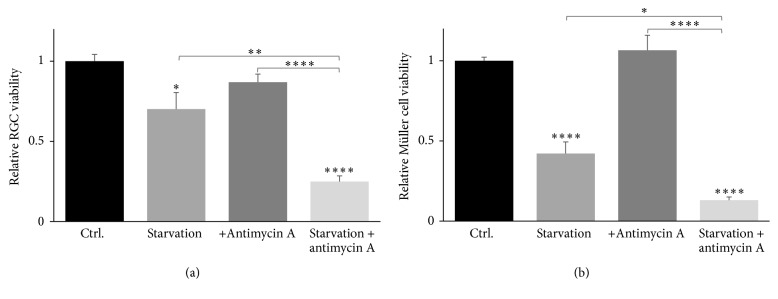
Cell viability of primary RGCs and primary Müller cells in response to starvation and mitochondrial inhibition. By means of LDH assays, starvation for 24 hours was shown to decrease RGC survival significantly to 70% ((a); ^*∗*^ *P* < 0.05, ^*∗∗*^ *P* < 0.01) compared to control. Likewise, starvation of Müller cells significantly decreased their survival to 42% of control ((b); ^*∗∗∗∗*^ *P* < 0.0001). Mitochondrial inhibition by exposure to 10 *μ*M antimycin A did not affect RGC and Müller cell survival ((a) and (b), resp.), whereas simultaneous starvation and exposure to antimycin A significantly reduced RGC and Müller cell survival to 25% and 13%, respectively ((a) and (b); ^*∗∗∗∗*^ *P* < 0.0001). Bars represent mean values ± SEM of 5–8 experiments. Differences in survival were tested using one-way ANOVA followed by Tukey's multiple comparisons test.

To evaluate the cytotoxic effects of starvation and mitochondrial inhibition by antimycin A on primary Müller cells and primary RGC cultures, we measured LHD levels. A significant decrease in RGC survival was found in response to 24 hours of starvation (Figure 3(a); 70% survival, *P* < 0.05). Simultaneous starvation and exposure to antimycin A cause a more pronounced RGC death (Figure 3(a); 25% survival, *P* < 0.0001), whereas RGC survival was unaltered in cells solely exposed to antimycin A. A more significant and pronounced decrease of Müller cell viability was found due 24 hours of starvation (Figure 3(b); 42% survival, *P* < 0.0001). As with RGCs, Müller cell viability was further reduced during simultaneous starvation and treatment with antimycin A (Figure 3(b); 13% survival, *P* < 0.0001). Antimycin A exposure did not affect Müller cell viability in cells with sufficient energy supply.

### 3.3. Glutamate Increases Survival of Starved Müller Cells

**Figure 4:**
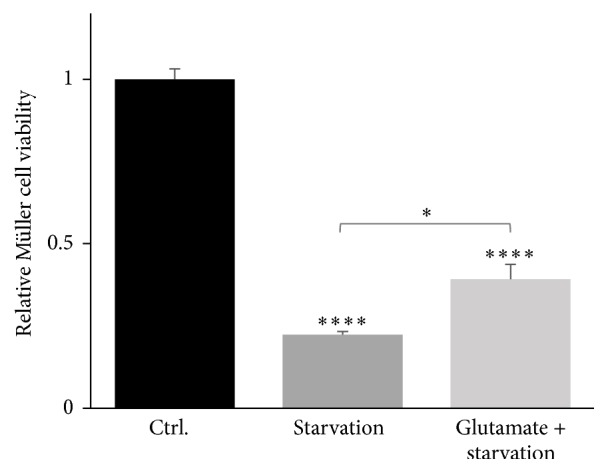
Cell viability of primary Müller cells in response to starvation and treatment with glutamate. By means of MTT assays survival of starved Müller cells significantly decreased to 22% (^*∗∗∗∗*^ *P* < 0.0001) compared with control. Additional treatment with 1 mM glutamate in combination with starvation also reduced the Müller cell viability to 39% (^*∗∗∗∗*^ *P* < 0.0001). Nevertheless, treatment with glutamate enhanced Müller survival significantly compared to solely starved Müller cells (^*∗*^ *P* < 0.05). The bars represent mean values ± SEM of 3 experiments. Differences in survival were tested using one-way ANOVA followed by Tukey's multiple comparisons test.

A significant decrease in Müller cell viability due to starvation was confirmed using a MTT-based cell survival assay (Figure 4; 22%, *P* < 0.0001). By adding 1 mM glutamate the cytotoxic effect as a result of starvation was diminished (22% versus 39%; *P* < 0.05), albeit it remained significantly reduced compared with the control (39%, *P* < 0.0001).

### 3.4. Starvation Increases Glutamate Uptake in Primary Müller Cells

**Figure 5:**
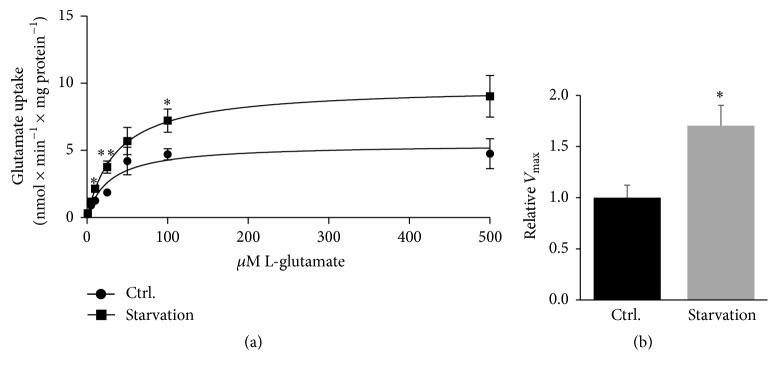
Glutamate uptake in starved Müller cells. Müller cells were cultured 24 hours in energy sufficient conditions or starved within the same time frame. Kinetic L-glutamate uptake at extracellular glutamate concentrations from 1 to 500 *μ*M was measured during the described conditions. Starvation significantly increased glutamate uptake ((a); ^*∗*^ *P* < 0.05 and ^*∗∗*^ *P* < 0.01. *P* = 0.067 at 500 *μ*M glutamate). The relative maximal uptake rate, *V*_max_, was also significantly increased ((b); *P* < 0.05). Values are represented as mean values ± SEM of 5 experiments. Differences were tested using unpaired two-tailed *t*-test at the different glutamate concentrations.

To determine the influence of starvation on the glutamate uptake into primary Müller cells, glutamate uptake was analysed in response to 24 hours of starvation at 7 different extracellular concentrations of glutamate from 1 *μ*M to 500 *μ*M. Our study revealed a significantly increased and consistent uptake of glutamate throughout the experiment in particular at extracellular glutamate concentrations of 10, 25, and 100 *μ*M glutamate (Figure 5(a); *P* < 0.05 and *P* < 0.01). Moreover, the relative *V*_max_ was significantly increased upon starvation (*P* < 0.05, Figure 5(b)).

### 3.5. Cocultures to Study Glia-Neuron Interaction

**Figure 6:**
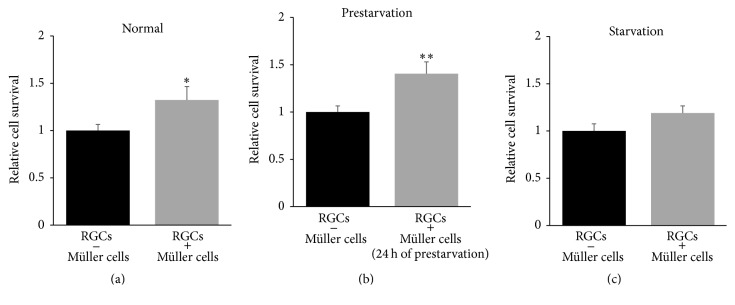
Müller cells increase RGC survival in cocultures. Importance of presence of Müller cells for RGC survival was investigated by coculturing of the primary cell cultures. The presence of energy sufficient Müller cells for 24 hours significantly increased RGC survival by 1.3-fold ((a); ^*∗*^ *P* < 0.05). A slightly increased RGC survival was found when Müller cells were prestarved for 24 hours before being inserted on top of the RGCs. In this condition the survival rate increased to 1.4-fold upon 24 hours of coculture ((b); ^*∗∗*^ *P* < 0.01). Finally, simultaneous starvation of RGCs and Müller cells in cocultures resulted in a tendency towards increased RGC survival ((c); 1.2-fold, *P* = 0.099). Bars represent mean values ± SEM of 4–9 experiments. Differences were tested using unpaired two-tailed *t*-test.

**Figure 7:**
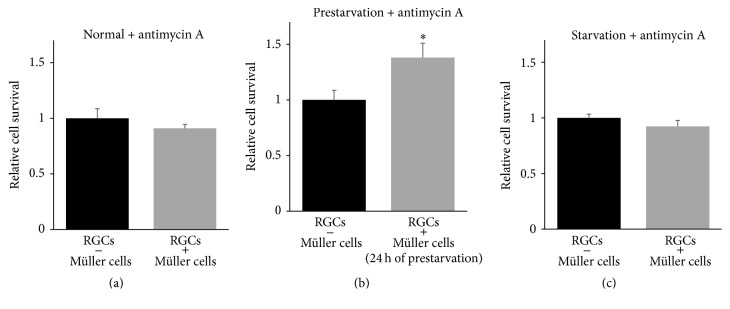
Prestarved Müller cells promote RGC survival during mitochondrial inhibition. RGC survival was analysed in cocultures with exposure to antimycin A (10 *μ*M) for 24 hours. During compromised mitochondrial function, the presence of Müller cells had a significant impact on RGC survival when the Müller cells were prestarved ((b); 1.4-fold, ^*∗*^ *P* < 0.05). The experiment showed no alterations of RGC survival in cocultures with sufficient energy supply (a) nor in cocultures with simultaneous starvation and treatment with antimycin A (c). Bars represent mean values ± SEM of 3–9 experiments. Differences were tested using unpaired two-tailed *t*-test.

RGCs were cultured in wells including an insert membrane containing primary Müller cells in close proximity, yet without anatomical contact. During this condition we found a significant impact on RGC survival in wells grown with Müller cells compared to RGCs cultured with an empty membrane insert (Figure 6(a); 1.3-fold, *P* < 0.05). When prestarving the Müller cells for 24 hours, we demonstrated significant increase of RGC survival after coculturing for 24 hours (Figure 6(b); 1.4-fold, *P* < 0.01). Moreover, we demonstrated a tendency towards an increased survival of RGCs in response to starvation (Figure 6(c); 1.2-fold, *P* = 0.099). To evaluate the effect of Müller cells on RGC survival when mitochondrial functions were compromised, we cultured RGCs and Müller cells in presence of antimycin A. During compromised mitochondrial function, the Müller cells had significant impact on the RGC survival due to 24 hours of prestarvation (Figure 7(b); 1.4-fold, *P* < 0.05). We found no protective effect of Müller cells on RGC survival in cells with sufficient energy availability after exposure to antimycin A. Similarly, no increased RGC survival was seen in starved cells exposed to antimycin A (Figures 7(a) and 7(c), resp.).

## 4. Discussion

RGC loss is a consequence of a variety of neurodegenerative diseases in the retina. Among the most common neurodegenerative retinal diseases are glaucoma and diabetic retinopathy [33–35]. Glaucoma is the most common cause of irreversible blindness [36, 37]. It encompasses several conditions that all lead to progressive loss of RGCs and their axons [38]. Diabetic retinopathy is another blinding disease caused by damage and visual loss [35]. Most neurodegenerative retinal diseases are age-dependent and the burden is increasing due to an aging population [36, 37]. In this matter, neuroprotective strategies have been increasingly studied [37]. No current strategies have shown promise yet. Since RGCs remain unmyelinated and are protected only by the surrounding macroglial cells, it has been suggested that glia-neuron partnership is essential for RGC survival. However, only limited evidence exists on glia-neuron interactions in the retina. One of the first contemporary studies to present glia-neuron interplay was performed by Turner and Cepko [39]. The study proposed that Müller cells perform many of the metabolic, ionic, and extracellular buffering requirements of neurons [39]. In the following years, an interesting concept of cooperability between retinal neurons and Müller cells was proposed by Reichenbach et al. [40]. Additional studies reported important roles of Müller cells in transporting energy sources from the vessels to the neurons [41] as well as removing glutamate from the extracellular space by uptake carriers [42]. Another implication of the importance of glia-neuron interaction is the secretion of factors that favor neuronal survival by Müller cells [43].

In present study, we emphasize the importance of this cellular relationship by coculturing primary RGCs with primary Müller cells. Furthermore, we aimed to investigate the consequences of starvation and inhibition of mitochondrial function by antimycin A on these cocultures. It is well established that hypoglycemia may lead to retinal neuronal loss* in vitro* [16–18]. The association between mitochondrial dysfunction and glaucoma has been reviewed [11, 44] and oxidative stress as a result of mitochondrial impairment has been connected to glaucomatous damage for several years [14]. We demonstrate that Müller cells increase RGC survival significantly during normal conditions and during starvation as well as prestarvation. Moreover, the presence of prestarved Müller cells promotes RGC survival during inhibited mitochondrial function. Since starvation causes decreased RGC and Müller cell survival the simultaneous increased ability of Müller cells to take up glutamate may indicate a protective mechanism that prevents glutamate-induced excitotoxicity. Altogether, our coculture model of primary RGCs and Müller cells confirms important roles of Müller cells in RGC survival. Previous studies have examined similar glia-neuron interactions. Furuya et al. demonstrated an increased survival rate in a coculture model of primary RGCs and Müller cells [27]. Moreover, Kawasaki et al. found a protective effect of primary Müller cells against glutamate excitotoxicity on primary RGCs [5]. A study by Matteucci et al. described Müller cell protection of RGCs against high-glucose conditions [28]. In fairness one study by Kashiwagi et al. showed opposite decreased RGC survival when cocultured with Müller cells [25]. All of the above-mentioned studies were performed with cell cultures originating from rats. To the best of our knowledge the present study is the first to describe the supporting role of Müller cells in RGC survival in primary cell cultures from mice as well as during prolonged starvation and mitochondrial inhibition, although the precise mechanism still needs elucidation. Previous studies examined the effect of various neurotrophic factors on RGC survival [30], including brain-derived neurotrophic factor (BDNF), which is produced by Müller cells in the retina [45]. BDNF is assumed to affect the survival of RGCs [46] and BDNF has been shown to upregulate GLAST and GS and furthermore to increase the glutamate uptake during hypoxia, of which the latter may accentuate the neuroprotective effects [47]. Pigment-epithelium-derived factor (PEDF) secreted by Müller cells is also contributing to the neuroprotective feature of Müller cells towards RGC survival [48]. An intravitreal PEDF transfection in a mouse model of glaucoma (DBA/2J mice) resulted in reduced loss of RGCs and the total nerve fiber layer, delayed vision loss, and reduced GFAP expression in the retina and optic nerve [49]. Ciliary neurotrophic factor (CNTF) has also been demonstrated to increase RGC survival after injury [46, 50]. Despite the fact that CNTF is not secreted by Müller cells, it has been suggested to modulate retinal glial cells into a more neuroprotective phenotype, which among others is characterized by a more efficient buffering of high concentrations of glutamate [51].

The glia-neuron partnership includes a range of decisive elements for the RGC homeostasis. Among these is the glutamate-glutamine cycle. In recent years, the focus on excitotoxicity [52, 53] has shifted from neuronal responses to glial regulations and responses to glutamate. Here, we demonstrate that Müller cells significantly increase their ability to take up glutamate during starvation. This finding extends our previous findings in the Müller cell line, MIO-M1, showing a similar increase in glutamate uptake during starvation [15]. However, we recently published a study demonstrating a diminished function of the glutamate uptake in Müller cells during simultaneous starvation and oxidative stress. These observations indicate a disturbed Müller cells homeostasis in presence of more than one environmental stressor [54]. Our coculture system provides a model to study glia-neuron interactions by permitting quantitative assessment of the effects of different stimuli on glial cells and neurons separately. Future implications of this system could allow manipulation of gene expression on only one cell type to enlighten the importance of specific proteins in the glia-neuron interplay.

The effects of 24 hours of starvation were evaluated by cell viability assays and surprisingly the experiments revealed greater vulnerability of Müller cells compared to RGCs. This observation indicates a greater robustness of RGCs to environmental stress such as starvation. This could be due to a more complex formulation of the ND-growth medium used for RGCs, whereas DMEM used for Müller cell cultures were not supplemented with various neurotrophic factors. Winkler et al. previously examined the viability of Müller cells upon 4 hours of starvation and the study showed a resistance of Müller cells to this relatively transient starvation [55]. A study by Wood et al. using a mixed retinal cell culture did indeed demonstrate that glial cells require a supply of glucose for survival in a culture upon 36 hours of starvation [56]. The sensibility of Müller cells upon starvation prompted us to analyse the Müller cell viability using another cell viability assay. Through MTT viability assays, the Müller cell viability was even further reduced due to 24 hours of starvation (Figure 4). Interestingly, the addition of glutamate to the starvation medium significantly increased the Müller cell survival indicating a capability of Müller cells to consume glutamate as an energy substrate. The oxidative metabolism of glutamate has been well described in brain astrocytes both* in vitro* and* in vivo* [57–59]. On the contrary, the use of glutamate as an energy substrate is less described in Müller cells [41, 60]. Additionally, the vulnerability of Müller cells due to starvation potentially suggests a further increase of glutamate uptake into Müller cell than the uptake experiment might indicate (Figure 5(a)). The uptake experiment was corrected for mg protein and therefore the correction does not necessarily reflect viable cells with a functional glutamate uptake.

In conclusion we show that Müller cells promote RGC survival independently of anatomical interaction. Hence, secreted factors as well as increased removal of excess extracellular glutamate may be essential in RGC-Müller cell interactions. Overall, our study indicates essential roles of Müller cells in RGC-related neurodegenerative conditions. Strategies aiming at regulating the homeostasis of Müller cells might prove useful in preventing RGC loss. Current studies in our laboratory are addressing glucose and glutamate metabolism within Müller cells in greater details. Future studies are necessary to further assess the potential of Müller cells as pharmacological targets in treatment strategies of RGC-related inner retinal conditions.
